# Microstructure Turbulence Measurement in the Northern South China Sea from a Long-Range Hybrid AUV

**DOI:** 10.3390/s23042014

**Published:** 2023-02-10

**Authors:** Yunli Nie, Xin Luan, Yan Huang, Libin Du, Dalei Song, Xiuyan Liu

**Affiliations:** 1 College of Ocean Science and Engineering, Shandong University of Science and Technology, Qingdao 266590, China; 2 College of Information Science and Engineering, Ocean University of China, Qingdao 266100, China; 3 Shenyang Institute of Automation Chinese Academy of Sciences, Shenyang 110016, China; 4 College of Engineering, Ocean University of China, No. 238 Songling Rd, Qingdao 266100, China; 5 Institute for Advanced Ocean Study, Ocean University of China, Qingdao 266100, China; 6 School of Information and Control Engineering, Qingdao University of Technology, Qingdao 266100, China

**Keywords:** ocean turbulence, hybrid AUV, dissipation rate, thermocline, mixing

## Abstract

This study describes the development of a long-range hybrid autonomous underwater vehicle (AUV) for ocean turbulence measurement. It is a unique instrument, combining the characteristics of the conventional AUV and the buoyancy-driven glider, with a variety of flexible motion modes, such as cruise mode, glider mode, drift mode, and combination of multiple motion modes. The hybrid AUV was used for continuous turbulence measurement in the continental slope of the northern South China Sea in 2020. A total of ten continuous profiles were completed covering a horizontal span of 25 Km and a depth of 200 m. The hybrid AUV was operated in the combined glider and cruise mode. The hybrid AUV’s flight performance was stable and satisfied the requirement for turbulence observation. The measured velocity shears from both probes were in good agreement, and the noise-reduced shear spectra were in excellent agreement with the Nasmyth spectrum. The water column in the study area was highly stratified, with a thick thermocline. The dissipation rate (ε) varied from 1.41 × 10^−10^ to 4.18 × 10^−7^ W·kg^−1^. In the surface mixed layer, high values of ε (10^−9^∼10^−8^ W·kg^−1^) were observed toward the water surface. In the thermocline, ε was 10^−9.5^∼10^−9^ W·kg^−1^, which was smaller than the level of the surface mixed layer. This result was mainly because of the strong “barrier”-like thermocline, which damped the transmission of wind and heat energy from the surface mixed layer to the deep layer. Overall, this study demonstrates the utility of hybrid AUVs for collecting oceanic turbulence measurements. They are a powerful addition to traditional turbulence instruments, as they make it possible to survey large areas to obtain high-quality and high-resolution data in both vertical and horizontal directions over long durations.

## 1. Introduction

In the spread of contaminants, sedimentation processes, and nutrient levels across all ocean zones, turbulent mixing and the resulting dissipation of energy are significant [1,2]. Consequently, it is essential to comprehend the distribution of turbulent energy under various background conditions.

Direct measurements of mixing require high-frequency measurements of variables such as current shear or temperature. According to Lueck et al. (2002) [3], a wide range of instruments for measuring vertical and horizontal turbulence have been developed. For instance, the vertical profiler, which is the instrument that is used the most, is a very quiet platform that does not have any mechanical vibration. The application of these instruments demonstrates the turbulence structure’s temporal and spatial variability. Vertical profilers, however, are limited in their ability to offer horizontal sampling because of the logistics involved in their deployment, especially in the upper ocean where horizontal inhomogeneity and the impact of phenomena such as Langmuir cells inside the mixed layer can be considerable. A range of instrument, including towed vehicles, submarines, AUVs and free-fall gliders, were used to measure horizontal turbulence beginning in the 1950s [4,5,6,7]. Because of their high deployment cost, early horizontal turbulence instruments were not widely used.

Recently, several small AUVs of 2–4 m length with shear probes have been developed to measure ocean turbulence, including the REMUS AUV, the glider, and others [8,9,10,11]. Their dissipation rates can be as low as 10^−11^ W·kg^−1^. The REMUS AUV has flexible mission types capable of accomplishing horizontal and vertical as well as bottom and surface observation tasks. It has been shown to outperform vertical profilers in investigating ocean mixed layers in the ocean under various conditions. As a result, using AUVs as a platform for measuring turbulence can result in long-term, continuous measurements in four dimensions. The measured data will help researchers investigate theories of turbulent cascade and stationarity in the oceans as well as comprehend the temporal and spatial variability of turbulent processes.

In this paper, a long-range hybrid AUV was developed to measure ocean turbulence in the northern South China Sea (*nSCS*). The hybrid AUV was a free-swimming, unmanned underwater vehicle that combined the attributes of the conventional AUV and the buoyancy-driven glider. This has made it possible to perform turbulent microstructural and meso-structural measurements that were previously not possible due to time, cost, or platform limitations. Multipass missions at various water depths in deep or shallow water enabled measurements in a variety of conditions within 1 m of the seafloor or sea surface. The methods are described in Section 2, with an emphasis on the design of the long-range hybrid AUV and the turbulence package. Section 3 describes the experiment, including the location and the sampling strategy. Subsequently, the results are presented in Section 4, including hybrid AUV flight performance, as well as local hydrography and microstructure data. Section 5 discusses the turbulent mixing characteristics of the *nSCS* and its relationship with the thermocline. Section 6 provides a brief conclusion of the study.

## 2. Methods

### 2.1. The Long-Range Hybrid AUV

**Figure 1:**
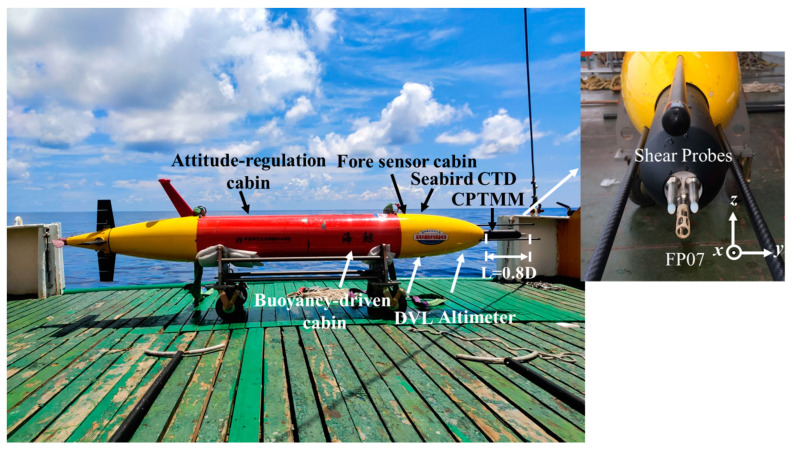
The structural components and oceanographic sensors of the long-range hybrid AUV. It is 3 m long, 0.35 m diameter, 200 kg mass and has a 1500 km endurance. Shown are the various micro and fine structure sensor systems. These include the Seabird GPCTD, the Nortek DVL, the dissolved oxygen, and the CPMTM for turbulence measurements. The CPMTM is in the middle of the fore sensor cabin and the shear probes are 0.8 D away from the nose.

**Figure 2:**
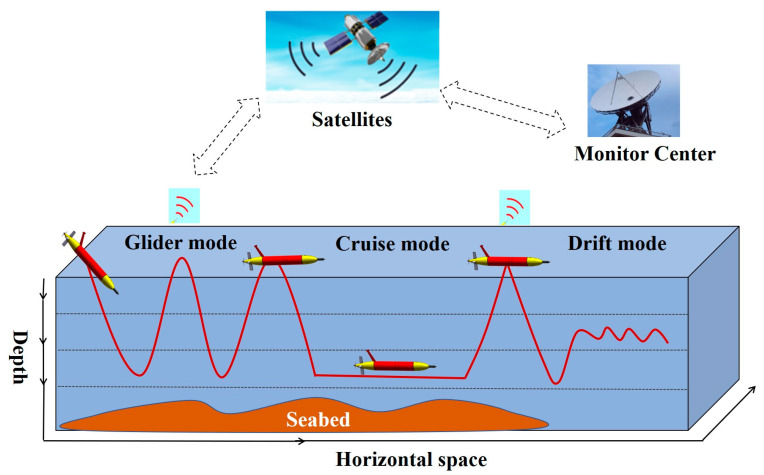
Typical working modes of the long-range hybrid AUV are: the glider mode, the cruise mode, and the drift mode. The long-range hybrid AUV combines the attributes of AUV and buoyancy-driven glider and has excellent maneuverability.

The long-range hybrid AUV is shown in Figure 1. It has a length of 3 m, a diameter (D) of 0.35 m and a mass of 200 kg [12,13]. For ocean surveys, the system has an endurance of 1500 km and can dive to 2000 m for oceanographic research. In terms of cruising capability, the vehicle is designed to reach up to 1.5 m/s at full speed. To execute different observation tasks with high efficiency, the design of the hybrid AUV combines the features of the glider and the conventional AUV. The buoyancy-driven cabin primarily features a fixed battery pack and a buoyancy control system that varies the net buoyancy of the system by adjusting the volume of the external oil bladder. The attitude-regulating cabin is designed to move the battery pack forward or backward to adjust the vehicle’s center of gravity. The propeller propulsion unit, the elevators, and the rudders make up the aft cabin. The elevators and the two rudders are coaxial. Therefore, the long-range hybrid AUV has a variety of flexible motion modes, such as yo-yo profiles similar to gliders (glider mode), cruising at a required depth (cruise mode), drifting with very little power consumption (drift mode), and combination of multiple motion modes, as shown in Figure 2.

A cross-platform instrument for microstructure turbulence measurements (CPMTM), a Seabird conductivity, temperature, and depth (CTD) sensor (with dissolved oxygen), a Doppler velocity log (DVL), and an altimeter were mounted on the hybrid AUV. All sensors were in the fore sensor cabin, which was near the bow (Figure 1). The low drag profile of the hybrid AUV was maintained while the data sampling accuracy was improved by separating the sensors from the rear propulsion system. Table 1 shows the performance parameters of the major sensors of the hybrid AUV. This array of sensors in a hybrid AUV enabled the quantification of important dynamic and kinematic turbulence and microscopic physical processes.

### 2.2. CPMTM

The CPMTM was an “all-in-one” payload with a length of approximately 0.6 m and a diameter of 0.08 m, and it had a flexible vibration-damping device inside it [14]. The performance of the instrument and the turbulent flow characteristics were both measured by the CPMTM’s numerous sensors. The cross-stream velocity variations (∂y⁄∂x) and the vertical velocity gradient (∂z⁄∂x) were measured using the shear probes [15], which were positioned orthogonally. The dissipation rate’s effective noise floor was discovered to be less than 10^−11^ W·kg^−1^ [14]. Microstructure temperature fluctuations were measured with a fast thermistor (FP07) [16], with a response time of approximately 7 ms. The CPMTM was fitted with a highly sensitive 3-axis accelerometer to measure the intensity of vibration during profiling. Shear signals could be post-processed to eliminate body vibrations. All turbulence channels (temperature and shear) were sampled at 1024 Hz, whereas the accelerometer channel was sampled at 512 Hz. The CPMTM was in the middle of the fore sensor cabin and the shear probes are 0.8 D away from the nose (Figure 1). 

## 3. Experiment

The South China Sea is the marginal sea of the Western Pacific Ocean, which is mainly composed of shoals, continental shelves, and deep-sea basins. Through the Luzon Strait, it is connected to the Western Pacific Ocean in its northeastern direction. The continental shelf break zone of the *nSCS* is very wide, and the terrain changes dramatically. Within fewer than 10 nautical miles, the water depth rapidly changes from 100 m to 1000 m. It is precisely because of the interaction between topography and tide that the activities of internal waves and internal tides in this region are very active and dissipated here, resulting in strong turbulent mixing [17].

**Figure 3:**
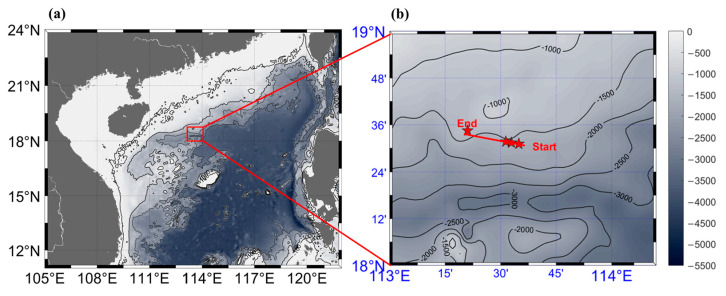
(**a**) Topography of the *nSCS* and the measuring area. (**b**) A larger image of the region enclosed by the red rectangle in panel (**a**), displaying the hybrid AUV path’s start and end points as well as the surrounding bathymetry. Color in both panels denotes the depth (m) of the water.

**Figure 4:**
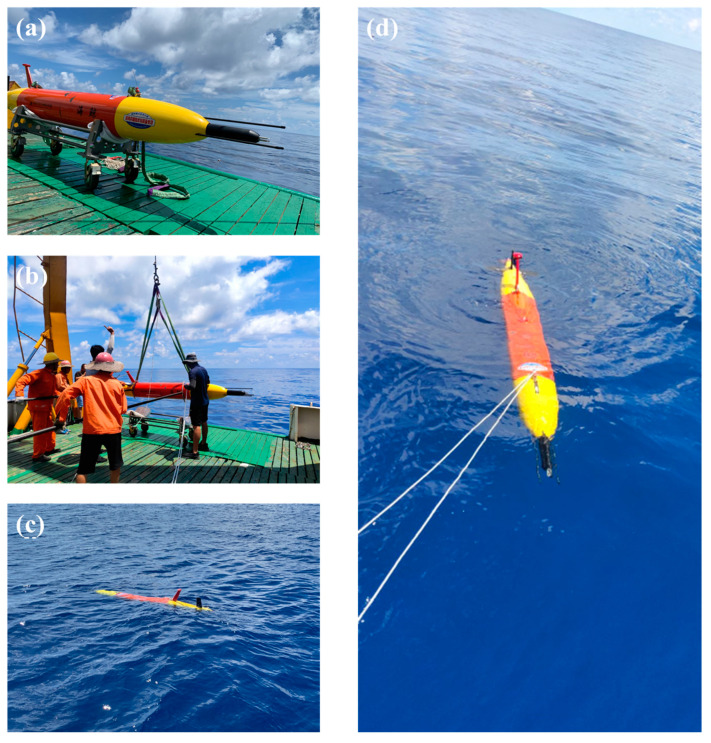
Deployment of the hybrid AUV from *R/V Yuezhanyuke* 8: (**a**) the hybrid AUV is ready on the deck, (**b**) the hybrid AUV is attached to the cables and lowered into the water through the A-frame of the *R/V*, (**c**) the hybrid AUV is released and starts work, and (**d**) the hybrid AUV back to the surface and retrieved.

From September 10th to 17th, 2020, A continuous and large-range turbulence measurement was performed on the slope of the *nSCS* aboard R/V *Yuezhanyuke 8*. Figure 3a depicts the measurement site within the *nSCS*, where the water depth was approximately 2000 m. Figure 3b displays the hybrid AUV’s precise track. From 06:00 to 17:00 on 14 September 2020 (local time), the hybrid AUV was observed along the 18°30′ N section from east to west. The operating mode was a combination of glider and cruise modes, with the glider mode having a pitch angle of about 13° between the surface and 200 m depth, and the cruise mode lasting 5 min at 200 m depth. Several high-quality spatiotemporal turbulence data of the upper ocean were collected as a result of the measurements, which produced a total of ten continuous profiles with a horizontal distance of 25 km. In the experiment, the hybrid AUV was used as the platform for our measurement, as shown in Figure 4.

## 4. Results

### 4.1. Flight Performance

The direction of the moving hybrid AUV relative to the horizontal is called the glide angle (*γ*), it is defined as the pitch angle (*θ*) plus the “angle of attack” (AOA or *α*):

(1) γ=θ+α,

The pitch angle (*θ*) is measured by the hybrid AUV’s attitude sensor, but the AOA is not measured directly. For high-quality dissipation rate calculation, crucial parameters such as the hybrid AUV speed through water (*U*) and the AOA need to be estimated as accurately as possible [18]. The AOA is obtained here using a hydrodynamic flight model developed by Merckelbach et al. (2010) [19], and uses measured pitch angle and pressure (*P*) to dynamically estimate *U,*

(2) $$U=\frac{W}{sin\left(\gamma \right)}=\frac{\partial P/\partial t}{sin\left(\theta +\alpha \right)}\;,$$

where *W = ∂P⁄∂t* is the AUV’s vertical speed based on the rate of pressure change.

**Figure 5:**
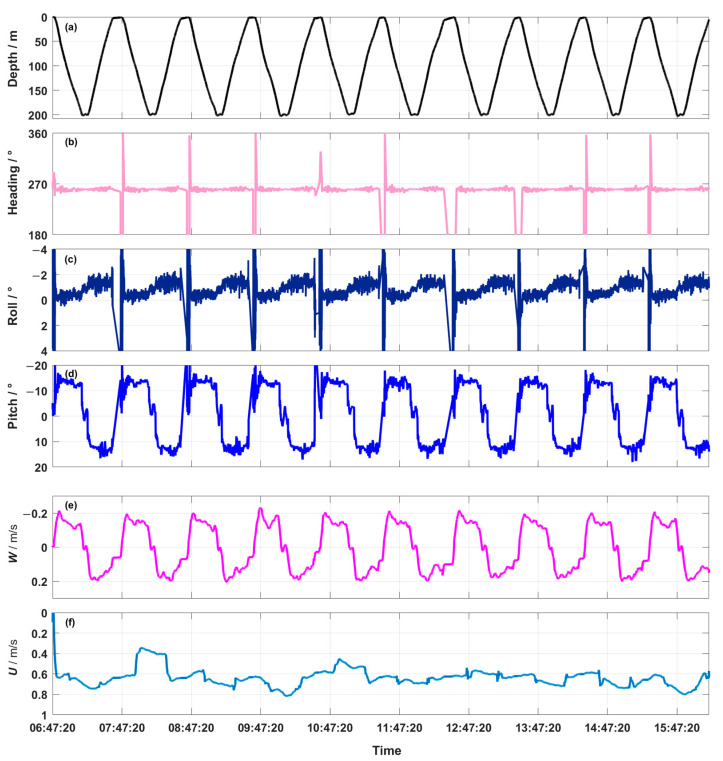
Flight performance of the hybrid AUV during the mission includes time series for (**a**) depth, (**b**) heading, (**c**) roll, (**d**) pitch, (**e**) *W*, and (**f**) *U*. The depth record shows the typical pattern with descent, cruise at 200 m, and ascent.

Figure 5 shows an overview of the data sampled by the hybrid AUV in the course of the mission. This includes time series for depth, heading, roll, pitch, vertical velocity, and *U*. In this mission, 10 continuous profiles were completed and the cruise mode of about 5 min was carried out at a depth of 200 m (Figure 5a). The flight path (Figure 5b) was a straight line with a constant heading of 270°. Figure 5c shows that the roll angle remained constant between 0° and 2°. During descent and ascent, the mean pitch angle was −13.42°/12.78° and the standard deviation was 1.31°/1.24° (Figure 5d). Near the interface, the roll and pitch variance substantially increased, the data of the upper 10 m was deleted. We were able to estimate the hybrid AUV’s speed along its flight path using the pitch angle and vertical speed (Figure 5e). Figure 5f shows that during descent/ascent, the mean speed of the hybrid AUV was 0.57 m·s^−1^/0.65 m·s^−1^, and in cruise mode was 0.68 m·s^−1^. The mean and standard deviation values of the hybrid AUV’s flight characteristics are summarized in Table 2. The hybrid AUV’s flight performance met the requirements for turbulence measurement and was stable.

### 4.2. Hydrography

**Figure 6:**
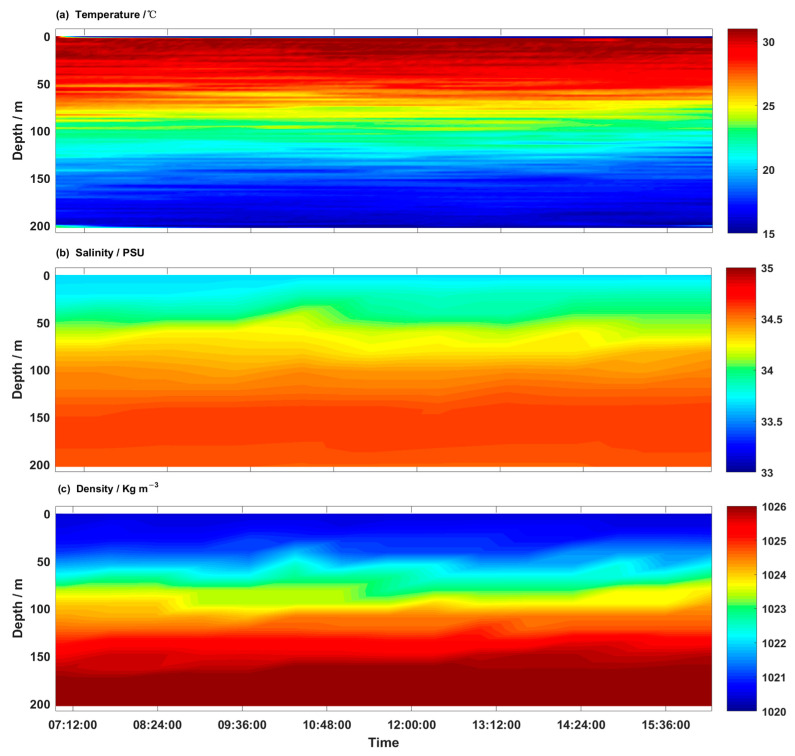
Contour plots of (**a**) temperature, (**b**) salinity, and (**c**) potential density measured by the hybrid AUV. According to the data, there was a thermocline between 40 and 150 m thick that separated the surface mixed layer and bottom mixed layer throughout the deployment.

Weather during the experiment was dominated by sunny conditions. The contour plots of temperature, salinity, and potential density along the section were depicted in Figure 6. The temperature’s vertical structure is depicted in Figure 6a. The sea surface temperature of the *nSCS* was about 30 °C, and the overall vertical temperature ranged from 14.9 °C to 30.7 °C. A prominent thermocline (40 m~150 m) and a thin surface mixed layer (~40 m) characterized the water column. The surface mixed layer is defined as the depth at which the temperature changes by 0.5 °C from the surface temperature [20]. In the thermocline, the temperature gradient is greater than 0.1 °C/m, and the temperature variation reaches ∆T ≈ 10 °C.

Salinity was 33.5 PSU at the surface and monotonically increased to almost 34.7 PSU below 150 m (Figure 6b). The density structure (Figure 6c) showed that throughout the deployment period, this region was strongly vertically stratified, with an average density difference of 5.9 kg·m^−3^ between the bottom and surface mixed layers. Figure 6a shows that the density structure was mainly controlled by temperature.

### 4.3. Microstructure Data

#### 4.3.1. Data Screening: Shear Probes

The shear probe voltage output was converted to velocity shear using the known shear probe sensitivity, electronic constant, flow through the sensor [3]. When the AUV maneuvers at the turn points, the angle of attack is large and the flow past the shear probe is almost zero. So, the velocity shear data is not available. In addition, when the AUV speed *U* > 0.4 m/s, the velocity shear data are valid. 

**Figure 7:**
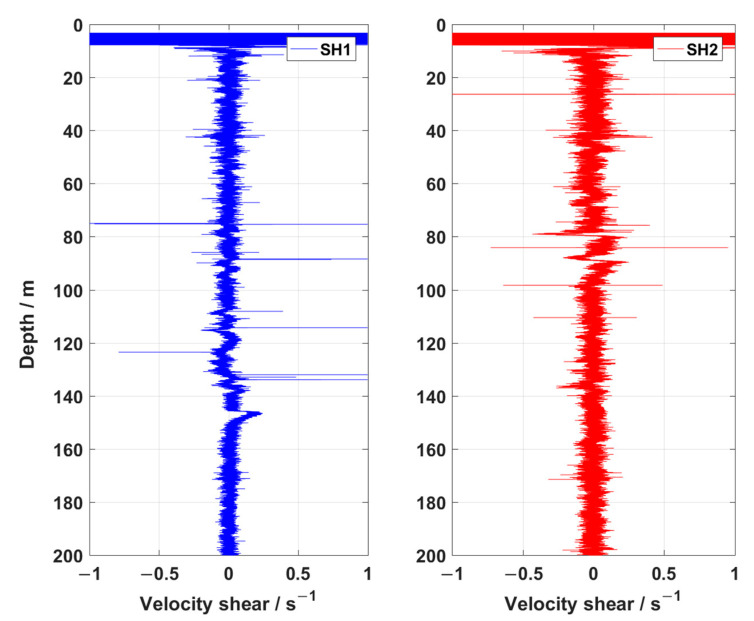
Sample of velocity shear of the two shear probes (SH1, SH2), collected during a steady descent of the fifth profile.

Therefore, firstly, data screening was carried out according to the AUV’s flight performance. Then, according to Rayda’s criterion, the singular data of the velocity shear signal were removed and replaced by the arithmetic mean value when the measurement error was three times the standard error [12]. Finally, the velocity shear signal was bandpass filtered from 0.15 Hz to 100 Hz to effectively remove the low-frequency motion and high-frequency vibration signature of the AUV. However, the elimination of this signal does not affect the dissipation rate calculation. A sample of velocity shear, collected during a steady descent of the fifth profile, is shown in Figure 7. Data in the surface were removed due to contamination by the flight performance of the AUV. After the start of the experiment, the velocity shear varied from −0.5 s^−1^ to 0.5 s^−1^, and they were very consistent.

#### 4.3.2. Shear Spectra

For spectral analysis, the velocity shear was divided into 12-s-long segment that were half-overlapping. We selected a fast Fourier-transform (FFT) length, corresponding to 4 s, detrend and Hanning window of each 4 s segment prior to calculating the spectra. The acceleration coherent noise was removed from the velocity shear signal using the method proposed by Goodman et al. [21] to reduce contamination from vehicle motion and vibration. For the best results and statistical significance when using the Goodman method, a record period that is longer than the FFT period is recommended. This method is based on the cross spectra between the shear probe and the accelerometer. We applied 12 s segments, which is three times the period of an FFT. The dissipation rate was calculated using the cleaned shear spectrum. Using Taylor’s frozen turbulence hypothesis and the *U*, the shear spectra *F*(*f*) in the frequency domain was transformed to the wavenumber domain (*k*), where *k* = *f*/*U* and *F*(*k*) = *UF*(*f*).

**Figure 8:**
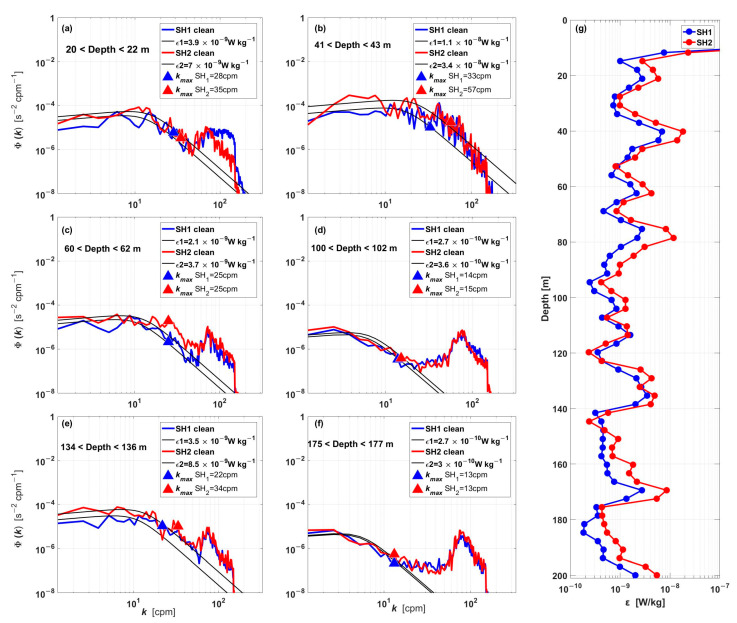
(**a**–**f**) Wavenumber shear spectrum samples from two shear probes (red and blue lines). The Nasmyth spectrum (black line) is shown for each case. Triangles (red and blue) mark the limits of $k_{max}$. The corresponding *ε* and depth are included in each plot. (**g**) The profile of *ε* calculated from the velocity shear.

Figure 8 shows the wavenumber spectra of the velocity shear in Figure 7 at different depths. It is clear that both shear probes’ measured wavenumber spectra (red and green lines) are in agreement with one another. They also match the corresponding Nasmyth spectrum well. These results demonstrate the high quality of the data and the hybrid AUV’s capacity to measure ocean turbulence.

#### 4.3.3. Estimation of Kinetic Energy Dissipation Rate

Assuming isotropic turbulence, the dissipation rate (*ε*) for each data set is calculated by integrating the wavenumber spectra as follows:

(3) $$\varepsilon_{i}=7.5v\bar{{\left(\frac{\partial u_{i}}{\partial x}\right)}^{2}}=7.5v\int_{k_{min}}^{k_{max}}\Phi_{u_{i}}\left(k\right)dk$$

where v represents the kinematic viscosity of seawater (≈ 1×10^−6^ m^2^·s^−1^), i (= 1, 2) is the number of shear probe, the overbar represents averaging, the$\;\Phi_{u_{i}}\left(k\right)$ is the estimated spectrum of velocity shear. The lower ($k_{min}$) and upper ($k_{max}$) integration limits of the spectrum are determined using Nasmyth spectrum for the turbulence [22], and the variance in the spectrum’s unresolved parts is corrected. We adopt the precise curve fit for the Nasmyth spectrum provided by Wolk et al. [23], when the shear spectrum is higher (lower) the Nasmyth spectrum, $k_{max}$ increases (decreases). According to the Nasmyth spectrum, integrating to 0.5$k_{k}$ where $k_{k}={\left(2\pi \right)}^{-1}{\left(\varepsilon /v^{3}\right)}^{1/4}$ is the Kolmogorov wavenumber, resolves 90% of the variance. 

Figure 8 shows some examples of wavenumber spectra and the corresponding estimates of *ε*. The shear spectra are depicted by the red curves, the Nasmyth spectrum fitted to the observed data are depicted by the black curves, and the $k_{max}$ limits are depicted by the green triangles. Figure 8g shows the profile of *ε* calculated from the velocity shear. The average value of *ε* was ∼10^−9^ W·kg^−1^.

## 5. Discussion

**Figure 9:**
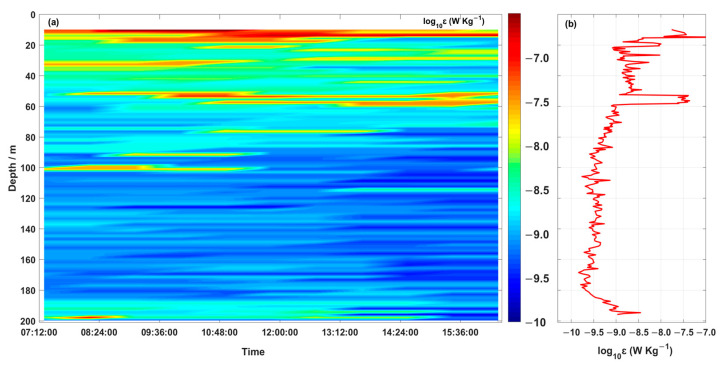
Depth–time maps of (**a**) the dissipation rate and (**b**) the temporally averaged dissipation rate.

Figure 9a shows the depth–time map of *ε*. Due to contamination from the hybrid AUV tilt, the data of the upper 10 m was deleted. The turbulence characteristics in this region are significantly different from those in shallow coastal waters, where turbulent mixing is strongly influenced by the thermocline. In the surface mixed layer, higher values of ε (10^−9^∼10^−8^ W·kg^−1^) were observed towards the water surface, mainly explained by the wind forcing. Variable dissipation was observed in the thermocline, with increased dissipation occurring at the upper boundary of the thermocline. The thermocline time-averaged *ε* ranges from 10^−9.5^ W·kg^−1^ to 10^−9^ W·kg^−1^ (Figure 9b), slightly less than the level of the open ocean thermocline (10^−9^ W·kg^−1^) [24,25]. Compared to the dissipation over the thermocline, the dissipation below it was weak, with time-averaged *ε* between 10^−10^ W·kg^−1^ and 10^−9.5^ W·kg^−1^ (Figure 9b). Dissipation rates were enhanced at the bottom boundary layer.

The intensity of diapycnal mixing produced by observations of turbulent kinetic energy dissipation is estimated using the Osborn relation for each *ε* estimate:

(4) $$K_{\rho }=\Gamma \;\varepsilon /N^{2},$$

where Γ is a dimensionless mixing efficiency, $N=\sqrt{\left(g/\rho_{0}\right)\left(\frac{d\rho }{dz}\right)}$ is the buoyancy frequency (here *g* and $\rho_{0}$ are the gravity and reference density, respectively, *ρ* is the potential density profile, and *z* is the depth). Here, we used the conventional constant of mixing efficiency, Γ = 0.2 [15,26].

**Figure 10:**
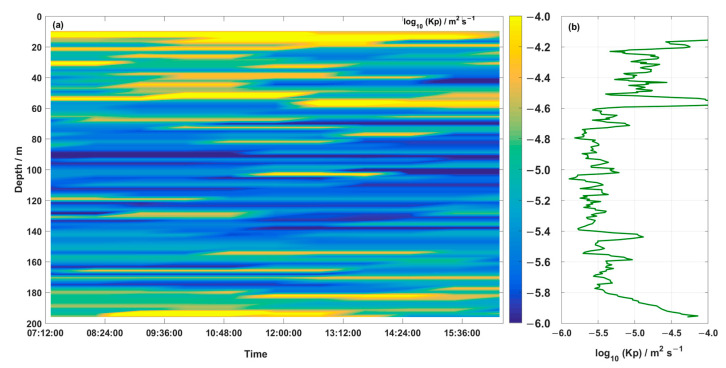
Depth–time maps of (**a**) diapycnal diffusivity and (**b**) the temporally averaged diapycnal diffusivity.

In the depth–time map of the diapycnal diffusivity in Figure 10a, a thin surface mixed layer is visible, with an averaged $K_{\rho }$ of 10^−5^ m^2^·s^−1^ and a strongly stratified thermocline with an averaged $K_{\rho }$ of 10^−5.5^ m^2^·s^−1^ (approximately 40 m~150 m). Below the thermocline, $K_{\rho }$ displayed the same pattern as *ε*. The periods of relatively low and high $K_{\rho }$ were well-matched with the periods of low and high *ε*, respectively (compare Figure 9b and Figure 10b), and the variations of $K_{\rho }$ were strongly correlated with the variations of *ε*.

The dissipation rate (*ε*) in this section varied from 1.41 × 10^−10^ to 4.18 × 10^−7^ W·kg^−1^. The larger turbulent energy dissipation occurred in the surface mixing layer, which was larger than that of the thermocline and bottom mixing layer by about three orders of magnitude. Due to the effects of sea surface wind energy and buoyancy flux, the vertical mixing of the surface mixing layer was large and uniform. In the upper boundary of the thermocline, there was a violent mixing process, and the change of turbulent energy dissipation rate and mixing rate was very obvious. With the increase in depth, the mixing below the thermocline gradually weakened, in the order of 10^−10^∼10^−9.5^ W·kg^−1^. Therefore, the thermocline was like a “barrier.” Above the “barrier” was an active open area, where wind energy and thermal radiation provided energy for strong turbulence mixing, whereas below the “barrier” turbulence mixing became very weak. The strong and stable thermocline in the water hindered the transfer of wind and heat energy from the surface mixed layer to the deep layer. The variation range of diapycnal diffusivity was 10^−6^~10^−4^ m^2^·s^−1^, and was higher between the surface mixing layer and the upper boundary of thermocline. These results are consistent with those of Shang et al. (2017) [27], who also found that the turbulent dissipation rate and diapycnal diffusivity would decrease under the thermocline “barrier.”

## 6. Conclusions

In this study, a hybrid long-range multi-motion AUV was developed and used to measure turbulence measurement in the *nSCS*. The hybrid AUV was operated in a combined glider and cruise mode to sample turbulence with high resolution in both the vertical and horizontal directions. A total of ten continuous profiles were completed covering a horizontal span of 25 km and a depth of 200 m. The hybrid AUV’s average speed (*U*) during descent and ascent was 0.57 m·s^−1^ and 0.65 m·s^−1^, respectively, while in cruise mode it was 0.68 m/s. The hybrid AUV’s flight performance was stable and satisfied the requirement for turbulence observation. The orthogonally installed shear probes recorded the velocity shear with good agreement, and the noise-reduced shear spectra from both probes were in excellent agreement with the Nasmyth spectrum. The lowest detection level of the dissipation rate was <3 × 10^−10^ W·kg^−1^, which is comparable to that of the majority of vertical microstructure profilers. 

The water column in the study area was highly stratified, with an average thick thermocline extending from 40 to 150 m below the surface. The dissipation rate (*ε*) in this section (Figure 9) varied from 1.41 × 10^−10^ to 4.18 × 10^−7^ W·kg^−1^. High values of *ε* (10^−9^∼10−^8^ W·kg^−1^) were observed in the surface mixed layer, pointing toward the sea surface. In the upper boundary of the thermocline, there was a violent mixing process, and the change of dissipation rate and diapycnal diffusivity was very obvious (Figure 9b and Figure 10b). In the thermocline, there was obvious variation in the dissipation rate and diapycnal diffusivity. The average dissipation rate and diapycnal diffusivity in the thermocline were 10^−9.5^∼10^−9^ W·kg^−1^ and 10^−5.5^ m^2^·s^−1^, respectively, which were smaller than the level of the surface mixed layer. Therefore, the thermocline was like a “barrier,” above which was an active open area, with wind energy and thermal radiation providing energy for strong turbulence mixing, and below which turbulence mixing became very weak. The strong and stable thermocline in the water damped the transfer of energy.

Overall, the measurement of turbulence from hybrid AUVs is a powerful addition to traditional turbulence instruments, as they make it possible to survey over long periods and large areas with high spatial resolution in both vertical and horizontal directions. Furthermore, the measurements are reasonably accurate and require much less dedicated time.

## Figures and Tables

**Table 1:** The hybrid AUV measured parameters, sensor type, and sensor specifications.

Parameter	Range	Precision	Resolution	Fs (Hz)
Current profiling	16 m/s	±0.1%/±0.1 cm/s	0.1 cm/s	8
Temperature	−5–42 °C	±0.002 °C	0.001 °C	
Conductivity	0–9 S/m	±0.0003 S/m	0.00001 S/m	1
Depth	2000 m	±0.1%/FS	0.002%/FS	
Dissolved oxygen	120% of saturation	±2% of saturation	/	1
Velocity shear	0–10 s^−1^	5%	10–3 s^−1^	1024
Fast temperature	−5–35 °C	0.005 °C	10–5 °C	1024
Accelerometer	±2 g	±1%	10–5 g	512

**Table 2:** Mean ± 1 standard deviation of the hybrid AUV flight parameters of the mission (Note. 𝜃 is the pitch angle, *W* is the vertical speed, and *U* is the speed through the water).

	*Pitch* (°)	*Vertical Speed* (m·s^−1^)	*U* (m·s^−1^)
Descent	−13.42 ± 1.31	−0.15 ± 0.02	0.57 ± 0.07
Ascent	12.78 ± 1.24	0.16 ± 0.02	0.65 ± 0.07

## Data Availability

Not applicable.

## Nomenclature

AUV	Autonomous underwater vehicle
*nSCS*	the northern South China Sea
CPMTM	Cross-platform instrument for microstructure turbulence measurements
CTD	Conductivity, temperature, depth
DVL	Doppler velocity log
R/V	Research vessel
AOA	Angle of attack
PSU	Practical salinity units
FFT	Fast-Fourier transform
*ε*	The dissipation rate
*θ*	The pitch angle
*γ*	The glide angle
*α*	The angle of attack
*U*	The hybrid AUV speed through water
*P*	Pressure
*W*	The vertical speed
*k*	The wavenumber
*Kρ*	The intensity of diapycnal mixing
